# Effects of prenatal polycyclic aromatic hydrocarbons and childhood material hardship on reading achievement in school-age children: A preliminary study

**DOI:** 10.3389/fpsyg.2022.933177

**Published:** 2023-01-04

**Authors:** Paige B. Greenwood, Jacob W. Cohen, Ran Liu, Lori Hoepner, Virginia Rauh, Julie Herbstman, David Pagliaccio, Amy E. Margolis

**Affiliations:** ^1^Department of Psychiatry, Vagelos College of Physicians and Surgeons, Columbia University, New York, NY, United States; ^2^Division of Child and Adolescent Psychiatry, New York State Psychiatric Institute, New York, NY, United States; ^3^Department of Environmental and Occupational Health Sciences, SUNY Downstate Health Science University, Brooklyn, NY, United States; ^4^Heilbrunn Department of Population and Family Health, Mailman School of Public Health, Columbia University, New York, NY, United States; ^5^Department of Environmental Health Sciences and Columbia Center for Children’s Environmental Health, Mailman School of Public Health, Columbia University, New York, NY, United States

**Keywords:** reading, material hardship, air pollution, child development, toxicants

## Abstract

**Background:**

Children from socioeconomically disadvantaged backgrounds are at elevated risk for reading problems. They are also likely to live in neighborhoods with high levels of air pollution and to experience material hardship. Despite these risk factors, the links between prenatal chemical exposures, socioeconomic adversities, and reading problems in youth from disadvantaged backgrounds remain understudied. Here we examine associations between prenatal exposure to polycyclic aromatic hydrocarbons (PAH), a common air pollutant, and reading skills, and determine if this relationship is exacerbated by material hardship among Black and/or Latinx children who have been followed as part of a longitudinal urban birth cohort.

**Methods:**

Mothers and their children, who were participants in a prospective birth cohort followed by the Columbia Center for Children’s Environmental Health, were recruited for the current study. Personal prenatal PAH exposure was measured during the third-trimester of pregnancy using a personal air monitoring backpack. Mothers reported their level of material hardship when their child was age 5 and children completed measures of pseudoword and word reading [Woodcock Johnson III Tests of Achievement (WJ-III) Basic Reading Index] at age 7. We used multiple linear regression to examine the effects of the interaction between prenatal PAH and material hardship on Basic Reading Index, controlling for ethnicity/race, sex, birthweight, presence of a smoker in the home (prenatal), and maternal education (prenatal) (*N* = 53).

**Results:**

A prenatal PAH × material hardship interaction significantly associated with WJ-III Basic Reading Index scores at age 7 (β = −0.347, *t*(44) = −2.197, *p* = 0.033). Exploratory analyses suggested that this effect was driven by untimed pseudoword decoding (WJ-III Word Attack: β = −0.391, *t*(44) = −2.550, *p* = 0.014).

**Conclusion:**

Environmental chemical exposures can be particularly toxic during the prenatal period when the fetal brain undergoes rapid development, making it uniquely vulnerable to chemical perturbations. These data highlight the interactive effects of environmental neurotoxicants and unmet basic needs on children’s acquisition of reading skill, specifically phonemic processing. Such findings identify potentially modifiable environmental risk factors implicated in reading problems in children from economically disadvantaged backgrounds.

## Introduction

Reading disorders (RD) affect 5–11% of school-age children, entailing impairment in single-word decoding, fluency, and reading comprehension (Shaywitz and Shaywitz, 2005; Snowling and Melby-Lervag, 2016). Genetics explain roughly 60% of the variance in RD among children living in socioeconomically advantaged settings, but this does not extend to children living in the context of economic disadvantage (Hensler et al., 2010; Peterson and Pennington, 2015). For these children, environmental factors appear to explain a larger portion of the variance in reading problems (Peterson and Pennington, 2015; Wodtke and Parbst, 2017). Although many household-level factors impacting reading skill acquisition have been examined, the role of the chemical environment, i.e., exposure to neurotoxicants, has been largely overlooked in research assessing reading problems (Margolis et al., 2020a; Margolis et al., 2021). Further, children living in the context of economic disadvantage are disproportionally at risk for such exposures given differential situating of the sources of toxic chemical exposures near lower income neighborhoods (Evans, 2004; Miranda et al., 2011; Bell and Ebisu, 2012; Hajat et al., 2015; Mikati et al., 2018; Jbaily et al., 2022). Thus, because of differential exposure to both toxic chemical and adverse social exposures, we have theorized that children from economically disadvantaged backgrounds will be at excess risk for developing environmentally associated phenotypes of reading problems.

Polycyclic aromatic hydrocarbons (PAH) are a class of air pollutants with known neurotoxicity that are produced by the incomplete combustion of organic materials such as fossil fuels, tobacco smoke, and burning of oil and coal for heat and/or electricity (Boström et al., 2002; Miller et al., 2004). Although air pollution is ubiquitous (Olden and Poje, 1995; Perera et al., 2002; Breysse et al., 2005), economically disadvantaged minority urban populations live in neighborhoods with relatively higher levels of pollution (Bell and Ebisu, 2012; Mikati et al., 2018; Jbaily et al., 2022), placing them at higher risk for adverse health and developmental outcomes (New York City Department of Health, 1998/1999; Claudio et al., 1999; Perera et al., 2002; Federico and Liu, 2003). In the context of the fetal programming theory (Padmanabhan et al., 2016), insults during the perinatal period such as exposure to air pollution could alter neurodevelopmental processes (Davis et al., 2019; Lertxundi et al., 2019) leading to neurological or psychiatric disorders in adulthood (Grandjean and Landrigan, 2014). Early life exposure to air pollution is associated with higher likelihood of poor performance on tests of math and reading (Grineski et al., 2020; Lu et al., 2021) and needing academic support services (Stingone et al., 2017) in childhood. These prior studies relied on area-level models to estimate exposure to air pollution rather than personalized exposure data or individually measured academic achievement tests. We recently reported that higher personal exposure to prenatal PAH exposure was associated with poorer performance on an individually administered measure of reading achievement in adolescence (Margolis et al., 2021). Here we examine associations between personally measured prenatal PAH and individually assessed reading skills in a different sample of younger children also living in the context of economic disadvantage.

Bronfenbrenner’s ecological systems theory argues that a child’s development is dependent upon a complex system of relationships that are incorporated into their environment longitudinally (Bronfenbrenner, 1979; Bronfenbrenner and Ceci, 1994; Bronfenbrenner and Evans, 2000). Proximal processes within the “microsystem” or immediate environment are a driving force for human development (Bronfenbrenner and Morris, 1998; Bronfenbrenner and Evans, 2000). In the context of reading acquisition, the development of knowledge and/or skills needed for reading could vary based on what resources are available in the home literacy environment (Crosnoe et al., 2010; Han and Neuharth-Pritchett, 2015; Burris et al., 2019). Early life stressors associated with living in poverty affect cognitive outcomes and academic performance for developing children (Yoshikawa et al., 2012). When examining health disparities among economically disadvantaged children, conventional measures of socioeconomic status (SES), such as parental education, household income, and parental occupation, have been used as predictors (Montgomery et al., 1996; Weinick et al., 1998; Flores et al., 1999). However, findings suggest that proximal measures of overall well-being, like material hardship, may be more sensitive predictors of health outcomes for economically disadvantaged children (Luthar and Cushing, 1999; Beverly, 2000; Ouellette et al., 2004). Black and Latinx children are more likely than White children to live in economically disadvantaged contexts and to experience high levels of material hardship in the form of unmet basic needs, such as inadequate housing, educational and nutritional resources (Vishnevetsky et al., 2015), making material hardship a potentially important factor to consider in models of reading skill acquisition.

Individuals are rarely exposed to a single chemical toxicant or social stressor, and individuals living in the context of economic disadvantage often experience multiple exposures. Such multiple exposures likely have combined effects on developmental outcomes *via* shared cognitive, behavioral, and neurobiological pathways (Lewtas, 1994; Weiss, 2000). Specifically, prenatal PAH exposure exacerbated the effects of early life stress on attention and thought problems in late childhood (Pagliaccio et al., 2020), as well as the effects of maternal stress on hippocampal volumes at age 8 (Margolis et al., 2022). Additionally, higher exposure to environmental tobacco smoke and material hardship was associated with cognitive deficits in the first 2 years of life in urban African American and Dominican youth (Rauh et al., 2004). Notably, the compounding effects of prenatal exposure to air pollution and early life stress on academic skill acquisition have not yet been examined.

In this study, we examine the impact of interactions between personally measured prenatal exposure to PAH and unmet basic needs at age 5 on reading skill at age 7. Given prior work, we hypothesized that higher material hardship would moderate the associations between higher exposure to prenatal PAH and poorer reading skill acquisition in Black and Latinx school-age children. In follow-up analyses, we explore how specific components of reading skill (word reading or pseudoword reading) contribute to any significant associations between exposures and performance on these individually administered reading measures.

## Materials and methods

### Participants

Fifty-three participants from a prospective longitudinal birth cohort followed by the Columbia Center for Children’s Environmental Health were included in the current study. These 53 children were old enough to complete the childhood neurocognitive visit and had available WJ-III Basic Reading Index, prenatal PAH, material hardship, and all covariates (see section “Statistical analyses”). The original cohort enrolled pregnant mothers from obstetrics and gynecology clinics at the New York Presbyterian Hospital and Harlem Hospital between 1998 and 2006 (Perera et al., 2006). Women between the ages of 18–35 were enrolled in the study if they did not use tobacco products or illegal drugs, were free of diabetes, hypertension, or known HIV, and pursued prenatal care in the 20th week of pregnancy. A second cohort enrolled the second born children of these women (total *N* = 131); participants in the current study were enrolled from this second sibling cohort. All participants identified as Black and/or Hispanic/Latinx and resided in Washington Heights, Central Harlem, or South Bronx areas of New York City. This study was approved by the Institutional Review Boards of Columbia University and New York State Psychiatric Institute; parents provided consent and children provided assent.

### Prenatal polycyclic aromatic hydrocarbons exposure assessment

Mothers wore an air monitoring backpack for 48 continuous hours during the third trimester of pregnancy and placed it beside their bed when they slept. The backpack contained a filter that collected airborne vapors, aerosols, and particulate matter < 2.5 micrometers (PM2.5) from which eight PAHs (benz[*a*]anthracene, benzo[*a*]pyrene, chrysene, benzo[*b*]luoranthene, benzo[*k*]luoranthene, indeno-[1,2,3-*cd*]pyrene, disbenz[*a,h*]anthracene, and benzo[*g,h,i*]perylene) were extracted and measured (ng/m^3^) at Southwest Research Institute (see Supplementary Table 1). The total of all eight PAHs was right skewed so the natural logarithm of the data was calculated to provide normal distribution for better fit to the data as done in prior work (Perera et al., 2003; Perera et al., 2012), see Supplementary material. The distribution of ln PAH values were mean = 0.59, SD = 0.72. PAH scores were transformed to standardized z-scores for analysis.

### Material hardship

A survey was given to the parent when their child was 5 years old to determine material hardship, i.e., unmet basic needs in the past year (Mayer and Jencks, 1989). This included eight questions about affording food, housing, clothing and health-care; response options for question 1 ranged from “very satisfied” to “very dissatisfied” and for the questions 2–8 were “yes” or “no” responses (see Table 1).

**TABLE 1:** Survey questions examining material hardship.

1. Think about where you live, the food you eat, and the things you can afford to do and buy. How do you feel about your overall living condition? Would you say?
a. Very satisfied
b. Somewhat satisfied
c. Neither
d. Very dissatisfied
2. In the last year, has there been a time when you and your family needed food but couldn’t afford to buy it?
a. Yes
b. No
3. In the last year, has there been a time when you couldn’t afford a place to stay, or when you couldn’t pay the rent?
a. Yes
b. No
4. In the last year, has your gas or electricity been turned off because you couldn’t afford to pay the bill?
a. Yes
b. No
5. In the last year, have you needed to buy any type of clothing for yourself or your family because you couldn’t afford to pay for it?
a. Yes
b. No
6. In the last year, has there been a time when you or a member of your family needed medicine or medical care but didn’t get the treatment because you couldn’t afford it?
a. Yes
b. No
7. Do you currently receive Medicaid?
a. Yes
b. No
8. Do you currently receive any type of public assistance?
a. Yes
b. No

This table display the survey questions completed by mothers to examine items of material hardship such as living conditions, food, housing, clothing, health-care, and public assistance at age 5. Survey questions were rescaled as 0–1 with higher scores representing higher levels of material hardship.

The responses were summed and rescaled as 0–1 with higher scores representing higher levels of material hardship. This continuous score was standardized and used as an independent variable and moderator in all analyses. One participant’s material hardship scaled score was three standard deviations from the mean (z-score > 3) and was winsorized to the next most extreme non-outlier value.

### Reading achievement

Reading skills were assessed during childhood (range 6–8 years old, mean age = 6.83, SD = 0.38) by a trained research assistant and checked for administration and scoring by a certified school psychologist and licensed psychologist. Single word and pseudoword reading were measured using the Woodcock Johnson Tests of Achievement-III (WJ-III) (Woodcock et al., 2000), specifically the Word Attack and Letter Word Identification subtests. The WJ-III Letter Word Identification subtest measures untimed single-word reading and requires the participant to read the words out loud. The WJ-III Word Attack subtest requires the participant to read aloud pseudowords (nonsense words) untimed. The WJ-III Word Attack and Letter Word Identification subtests provide age-adjusted standard scores that comprise the weighted-norm Basic Reading Index, measuring untimed decoding abilities.

Children also completed the Test of Word Reading Efficiency-II (TOWRE-II), Site-Word Efficiency (SWE), and Phonetic Decoding Efficiency (PDE) (Torgesen et al., 2012). TOWRE-II is a timed reading assessment that measures the participant’s ability to read a list of words (SWE) and pseudowords (PDE) aloud for 45 s each. The SWE and PDE subtests are age-adjusted standard scores combined as a weighted-norm Word Reading Efficiency Index measuring timed decoding abilities.

### Statistical analyses

All statistical analyses were performed in IBM SPSS Statistics version 26. Distributions of key variables were assessed to address assumptions of normality in parametric tests (see Supplementary Figures 1, 2). The association between prenatal PAH and material hardship was assessed using Pearson correlation. In primary analyses, multiple linear regressions examined whether interactions between prenatal PAH and moderator material hardship were associated with untimed WJ-III Basic Word Reading Index (*N* = 53). Follow-up analyses examined if any significant results were driven by effects on the Word Attack or Letter Word Identification subtests that comprise the Basic Reading Index. PROCESS macro v4.0 for SPSS (Hayes, 2022) was used to determine the conditional effects of prenatal PAH exposure at values of material hardship on WJ-III reading measures. To test the specificity of material hardship in the primary model, control analyses evaluated the interaction between prenatal PAH and maternal education, an alternate measure of socioeconomic advantage, on reading outcomes. Exploratory analyses examined if the interaction between prenatal PAH and material hardship was associated with TOWRE-Word Reading Efficiency Index, which measures timed reading efficiency.

All models controlled for potentially confounding variables, including ethnicity/race (binary: Black or Latinx/Hispanic), sex, birthweight (grams), presence of smoker in the house (binary: yes or no) at prenatal visit, and maternal highest degree of education (categorical: less than high school to 4+ years of college) at prenatal visit. Control analyses examining the prenatal PAH exposure by maternal education interaction term included material hardship as a covariate. Two birthweight values were identified as outliers (z-score > 3) and were winsorized to the next extreme non-outlier value. Age was not included as covariate in the main analysis because the outcome variables (reading measures) were age-adjusted using standardized norms. A sensitivity analysis included age as a covariate. Johnson–Neyman plots were generated in RStudio v.3.5.1 to visualize values of the moderator for which the slope of the predictor is significant (*p* < 0.05). Complete case analyses of the data are presented. All tests were two-tailed, and significance thresholds were set at *p* < 0.05. All linear regression models were checked for normal distribution of residuals (see Supplementary Figure 3).

## Results

### Participants

Table 2 presents demographic data for the children included in this study. Fifty-four percent of the children identified as Dominican/Latinx and 46% identified as Black/African-American. Relative to children without complete data, a greater percentage of those included in the study were female. Relative to national norms (mean = 100, SD = 15), children’s reading scores were in the average range: WJ-III Basic Reading Scores (mean = 104.11, SD = 13.63); TOWRE Word Reading Efficiency Index scores (mean = 93.06, SD = 14.42). The range of observed values for z-scored material hardship were −1.10, 2.93 and for prenatal PAH were −1.95, 1.95. There were no significant associations between prenatal PAH and material hardship (*r* = −0.001, *p* = 0.99).

**TABLE 2:** Participant demographics.

	Participants with prenatal PAH, material hardship and Basic Reading Index (*n* = 53)	Participants without prenatal PAH, material hardship and Basic Reading Index (*n* = 78)	Group comparisons
Characteristic	Mean (SD) or *N* (%)	Mean (SD) or *N* (%)	
Ln total PAH (prenatal)	0.59 (0.72)	0.55 (0.93)	*t* = −0.1945, *p* = 0.85
Material hardship at age 5	0.30 (0.17)	0.37 (0.21)	*t* = 1.812, *p* = 0.07
Birthweight (grams)	3322.21 (330.35)	3448.58 (485.20)	*t* = 1.51, *p* = 0.13
Sex (% female)	45 (66.0%)	16 (41.0%)	χ^2^ = 5.690, *p* = 0.02*
Smoker in the house (% yes, prenatal)	11 (20.8%)	10 (12.8%)	χ^2^ = 1.476, *p* = 0.22
Ethnicity/Race (% Dominican)	32(60.4%)	44 (56.4%)	χ^2^ = 0.204, *p* = 0.65
Maternal education (prenatal)			G^2^ = 10.65, *p* = 0.16
Less than HS	0.0%	3(3.8%)	
Some HS	10(18.9%)	14(17.9%)	
HS diploma	8(15.1%)	19(24.4%)	
GED	2(3.8%)	6(7.7%)	
Some college	18(34.0%)	12(15.4%)	
2 year college degree	9(17.0%)	13(16.7%)	
4 year college degree	4(7.5%)	9(11.5%)	
4+ years of college	2(3.8%)	2(2.6%)	

This table displays demographic data for children included in the current study (*n* = 53) and those not yet old enough to complete the childhood neurocognitive visit (*n* = 78). Ln total PAH, natural logarithm of total polycyclic aromatic hydrocarbon; HS, high school; GED, general educational development; SWE, site-word efficiency; PDE, pseudoword decoding efficiency. *p < 0.05.

### Effects of prenatal polycyclic aromatic hydrocarbon and material hardship on reading achievement

**FIGURE 1:**
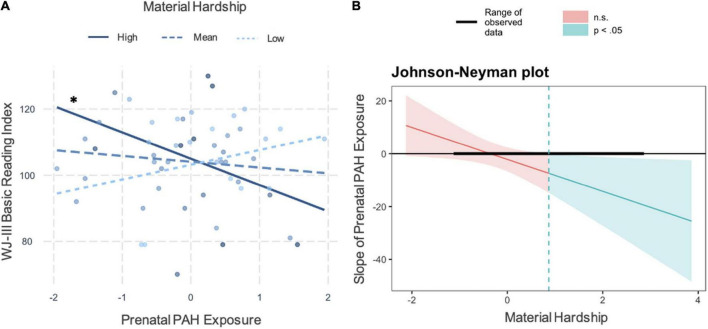
Interaction plot showing prenatal PAH by material hardship predicting *WJ-III* Basic Reading Index. **(A)** The prenatal PAH by material hardship interaction associated with lower Basic Reading Index at child age 7. Higher PAH associated with lower Basic Reading Index scores at higher levels of material hardship (mean ± 1SD), *p < 0.05. **(B)** Johnson–Neyman plot showing the slope of the association between prenatal PAH and Basic Reading Index at age 7 (*y*-axis). PAH was z-scored *here* (*M* = –0.05, SD = 0.90) so the slope can be interpreted as a 1 SD increase in PAH is associated with a Y unit change in Basic Reading Index scores. The observed range of material hardship z-scores was –1.10, 2.83 which is noted in black by the bold line at *y* = 0. The region of significance analysis indicates *p* < 0.05 significant association between prenatal PAH and Basic Reading Index in the shaded blue region, (no significant associations in the red, at values of material hardship between –3.18, 0.87). *represents a significant slope.

The prenatal PAH by material hardship interaction term was significantly associated with lower WJ-III Basic Reading Index (β = −0.347, *t*(44) = −2.197, *p* = 0.033, Figure 1A and Table 3). Higher prenatal PAH was significantly associated with lower WJ-III Basic Reading Index when material hardship values were elevated (z-score > 0.87, raw > 0.45; Johnson–Neyman plot, Figure 1B). For every 1 point increase in prenatal PAH exposure at high levels of material hardship, there is a 7-point decrease in WJ-II Basic Reading Index (see Supplementary Table 2). Including age as a covariate did not change these results (see Supplementary Table 4).

**TABLE 3:** Regression analyses predicting WJ-III Basic Reading Index.

*N* = 53	WJ-III Basic Reading Index		
	
	β	*t*	*P*
Ethnicity/Race	0.076	0.555	0.582
Maternal education (prenatal)	–0.044	–0.275	0.785
Sex	–0.082	–0.566	0.574
Birth weight (grams)	–0.299	–1.974	0.055
Smoker in the house (prenatal)	–0.015	–0.093	0.926
Ln total PAH (prenatal) – Z-scored	–0.139	–0.961	0.342
Material hardship at age 5 – Z-scored	0.066	0.417	0.679
PAH × Material hardship*	–0.347	–2.197	0.033

WJ-III Basic Reading Index is the dependent variable in the regression analyses. All models control for ethnicity/race, maternal education (prenatal), sex, birthweight (grams), and smoker in the house (prenatal). Regression coefficients (β) and their corresponding t-statistic and *p*-values are presented for all predictors in the model.

*p < 0.05. Ln total PAH, natural logarithm of total polycyclic aromatic hydrocarbon.

**FIGURE 2:**
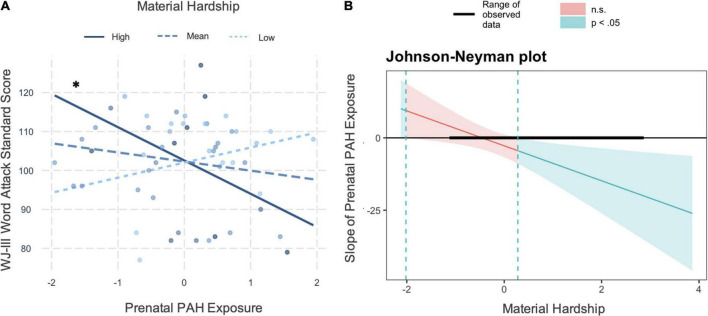
Interaction plot showing prenatal PAH by material hardship predicting WJ-III Word Attack standard scores. **(A)** The prenatal PAH by material hardship interaction associated with lower Word Attack standard scores at age 7. Higher PAH associated with lower Word Attack standard scores at higher levels of material hardship (mean ± 1SD), *p < 0.05. **(B)** Johnson–Neyman plot showing the slope of the association between prenatal PAH and Word Attack standard scores at age 7 (*y*-axis). PAH was z-scored here (*M* = –0.05, SD = 0.90) so that the slope can be interpreted as a 1 SD increase in PAH is associated with an Y unit increase in Word Attack values. The observed range of material hardship values was –1.10, 2.83 which is noted in black by the *y* = 0 line. The region of significance analysis indicates a *p* < 0.05 significant association between prenatal PAH exposure and Word Attack standard scores in the shaded blue region, (no significant associations in the red, at values of material hardship between –2.02, 0.28). *represents a significant slope.

In follow-up analyses, the prenatal PAH by material hardship interaction was significantly associated with pseudoword decoding (WJ-III Word Attack: β = −0.391, *t*(44) = −2.550, *p* = 0.014, Figure 2A and Supplementary Table 3). Higher prenatal PAH was significantly associated with lower pseudoword decoding when material hardship values were higher (z-score > 0.28, raw > 0.35; Johnson–Neyman plot, Figure 2B). For every 1 point increase in prenatal PAH exposure at high levels of material hardship, there is a 7-point decrease in WJ-II Word Attack scores (see Supplementary Table 5). This association was not significant at lower values of material hardship. In control analyses, the prenatal PAH by maternal education interaction term on Basic Reading Index was not significant (β = 0.723, *t*(44) = 1.709, *p* = 0.095). In an exploratory analysis, the prenatal PAH by material hardship interaction term on the TOWRE Word Reading Efficiency Index was not significant (β = −0.288, *t*(40) = −1.697, *p* = 0.097, *n* = 49).

## Discussion

The current study aimed to investigate if interactions between early life chemical and social exposures impact the development of reading outcomes for Black and Latinx children. Prior studies have separately examined the contributions of air pollution (Stingone et al., 2017; Grineski et al., 2020; Lu et al., 2021) and social adversities (Noble et al., 2006a; Dolean et al., 2019) to academic achievement. Here we show for the first time that prenatal exposure to PAH and material hardship have interactive effects on reading skill acquisition, and that such effects appear to be driven by pseudoword reading. Consistent with prior findings that more proximal versus conventional measures of SES provide greater insight into health disparities, we report that material hardship, and not maternal education, interacts with prenatal PAH to affect children’s reading skills. Critically, identifying material hardship as a moderator of children’s acquisition of reading skill provides a specific and modifiable target for prevention and intervention.

High exposure to both PAH and material hardship were associated with lower word reading scores. Word reading relies on decoding abilities through knowledge of grapheme-phoneme correspondence (Simos et al., 2002). The process of decoding words is supported by executive functions such as inhibitory control (Margolis et al., 2020b) which allows individuals to suppress prepotent responses and avoid guessing an orthographically similar word based on incomplete phonological information (Seidenberg et al., 1984; Messer et al., 2016). Childhood inhibitory control is vulnerable to the effects of environmental air pollution (Chiu et al., 2016; Margolis et al., 2016; Guxens et al., 2018) as well as poverty (Raver et al., 2013; Lawson et al., 2018). Thus, the compounding effects of prenatal air pollution exposure and unmet basic needs could impact the ability to reject irrelevant stimuli during word reading, resulting in poorer word recognition. Such findings are consistent with our prior work showing childhood inhibitory control as a mediator of pollution-related effects on adolescent reading comprehension (Margolis et al., 2021). Since reductions in word reading automaticity may underlie cognitive interference that disrupts reading comprehension (Lyon et al., 2003), pollution-related effects on emerging word reading skills observed in young children could develop into an adolescent phenotype characterized by difficulty with comprehension.

Alternatively, word reading problems might derive from deficits in automaticity and fluency rather than from decoding problems (Wolf and Bowers, 1999), as proposed in the double deficit hypothesis of reading. In this model, rapid automatized naming (RAN) underlies word reading accuracy and early reading fluency (Norton and Wolf, 2012; Araújo et al., 2015). Such processes are thought to rely on processing speed (Georgiou et al., 2013), which is a target of exposure to air pollution (Peterson et al., 2015) and poverty (Zeki Al Hazzouri et al., 2017). Thus, the observed effects of exposure on word reading could derive from the combined effects of exposure to air pollution and material hardship on processing speed, ultimately influencing word reading. Future studies should examine processing speed and inhibitory control as potential cognitive mediators of this environmentally associated phenotype of reading problems. In support of this hypothesis, the neural correlates that underlie these proposed cognitive mediators have been shown to be vulnerable to both air pollution exposure (Peterson et al., 2015; Guxens et al., 2018) and social adversities (Kishiyama et al., 2009; Zheng et al., 2022).

The combined effect of prenatal PAH exposure and childhood material hardship on word reading was driven by performance on pseudoword decoding. When reading pseudowords, a child is required to use knowledge of grapheme to phoneme conversion to serially decode unfamiliar non-words (Ouellette and Beers, 2008). The combined effects of environmental air pollution and unmet basic needs may impact phonological decoding *via* alterations to the left-lateralized reading circuit (Richlan et al., 2009; Richlan, 2012) or to circuits distinct from those typically identified in poor readers from higher SES backgrounds (Noble et al., 2006b). Both word and pseudoword reading engage the left occipital fusiform cortex (Devlin et al., 2006) which stores abstract visual word forms (Price, 2000). Activation in this region during a reading-related task was sensitive to sociodemographic factors such that brain-behavior associations were attenuated in children from higher but not lower SES backgrounds (Noble et al., 2006b). Specifically, despite poor phonological ability, children from lower SES backgrounds engaged regions involved in visual word recognition whereas children from higher SES did not. Such findings support our premise that an environmentally associated phenotype of reading problems may derive from effects of exposure on brain circuits not typically identified in children from higher SES backgrounds who have reading problems. Future studies should examine the impact of these exposures on brain functioning during reading-related tasks.

Black and Latinx children disproportionately live in economically disadvantaged contexts that include lower income neighborhoods with greater concentrations of air pollution (Bell and Ebisu, 2012; Mikati et al., 2018; Jbaily et al., 2022). The distribution of total PAH in our sample was similar to observed levels of toxicity in prior work showing that participants with higher exposure to prenatal PAH (relative to the rest of the sample) moderated the associations between maternal perceived stress and hippocampal volume (Margolis et al., 2022) and was associated with worse reading comprehension (Margolis et al., 2021). Minoritized children living in socioeconomically disadvantaged contexts are also more likely to experience material hardship in the form of housing, health, and food insecurities (Vishnevetsky et al., 2015). Here we examined unmet basic needs at age 5, which is a developmental time point when children are building the foundation for word reading through exposure to books and shared reading with their parent/caregiver (Puglisi et al., 2017). Thus, higher levels of material hardship in the early years may represent a pathway through which children are unable to develop these foundational reading competencies. Further, higher material hardship could increase parental distress and depression (Ashiabi and O’Neal, 2007; Gershoff et al., 2007; Zaslow et al., 2009), and affect positive parenting practices such as attending museums or providing access to books (Gershoff et al., 2007; Zaslow et al., 2009). Such pathways to academic disparities have important public health implications and identify potentially modifiable targets for supporting the overall well-being and academic achievement of Black and Latinx children.

Our study has some limitations. Given the sample size, these findings should be viewed as preliminary. Future studies with larger samples and longitudinal measurement of postnatal PAH exposure may improve our understanding of these trajectories. Our data do suggest that children living in economic disadvantage may be at increased risk for environmentally associated phenotypes of reading problems. We controlled for confounding variables such as ethnicity/race, sex, birthweight, presence of smoker in the house, and maternal years of education at prenatal visit; however, we may not be accounting for other variables that contribute to word reading variability such as the nature of parent-child reading interactions (Demir et al., 2011) or classroom instruction (Cameron et al., 2008). Our dataset does not include a school quality variable; this important variable should be considered and collected in future studies. In addition, our dataset comes from a sample of women and children from economically and racially disadvantaged backgrounds, where high levels of toxic environmental exposures further contribute to learning problems in these populations.

In conclusion, our findings suggest that children exposed to neurotoxicants such as air pollution and material hardship could manifest word reading problems by school-age. This environmentally associated phenotype of reading problems may represent the beginning of a negative cascade of events ultimately leading to altered reading comprehension skills in adolescence, as we have shown in prior work (Margolis et al., 2021). Structural factors that marginalize people of color into living in lower income neighborhoods with higher levels of toxic chemical exposures suggest a specific pathway by which academic disparities in children of color may arise, and some of these conditions are difficult to address, requiring a commitment to geopolitical change. The National Center for Education Statistics shows that economically disadvantaged children lag behind their peers on tests of reading achievement at 4th and 8th grade, calling attention to a steady achievement gap (NCES, 2020). Herein, our findings highlight the need to include a longitudinal assessment of reading outcomes to identify modifiable targets for intervention and potential prevention of environmentally associated phenotypes of reading problems. These data also show a need for scientific evidence to be incorporated into public policy to create sustainable change and address environmental health disparities that greatly impact child development. Critically, we note that legislation to reduce air pollution in New York City has led to declines in exposure which could translate to reductions in health risks (Lovasi et al., 2022). In the immediate future, our work may drive public health regulatory activity with an important impact on the reduction of these neurotoxic environmental exposures that contribute to the pathway, thereby protecting disadvantaged children from further harm. In addition, raising awareness in economically disadvantaged communities about the harmful effects of environmental pollutants could spark scientific and community centered partnerships to fight these growing disparities.

## Data availability statement

The original contributions presented in this study are included in the article/Supplementary material, further inquiries can be directed to the corresponding author.

## Ethics statement

The studies involving human participants were reviewed and approved by the Institutional Review Boards of Columbia University and New York State Psychiatric Institute. Written informed consent to participate in this study was provided by the participants’ legal guardian/next of kin.

## Author contributions

PG, JC, DP, RL, and AM performed the statistical analyses and data interpretation. AM, JH, VR, and LH performed the data collection with the Columbia Center for Children’s Environmental Health. All authors wrote, reviewed, edited, and approved the final manuscript.

## References

[B1] Araújo S. Reis A. Petersson K. M. Faísca L. (2015). Rapid automatized naming and reading performance: A meta-analysis. *J. Educ. Psychol.* 107 868–883. 10.1037/edu0000006

[B2] Ashiabi G. S. O’Neal K. K. (2007). Household Food insecurity predictive of health status in early adolescence? A structural analysis using the 2002 NSAF data set. *Calif. J. Health Promot.* 5 76–91. 10.32398/cjhp.v5i4.1269

[B3] Bell M. L. Ebisu K. (2012). Environmental inequality in exposures to airborne particulate matter components in the United States. *Environ. Health Perspect.* 120 1699–1704. 10.1289/ehp.1205201 22889745 PMC3546368

[B4] Beverly S. G. (2000). Using measures of material hardship to assess well-being. *Focus* 21 65–69.

[B5] Boström C. E. Gerde P. Hanberg A. Jernström B. Johansson C. Kyrklund T. (2002). Cancer risk assessment, indicators, and guidelines for polycyclic aromatic hydrocarbons in the ambient air. *Environ. Health Perspect.* 110 451–488. 10.1289/ehp.110-1241197 12060843 PMC1241197

[B6] Breysse P. N. Buckley T. J. Williams D. Beck C. M. Jo S. J. Merriman B. (2005). Indoor exposures to air pollutants and allergens in the homes of asthmatic children in innercity Baltimore. *Environ. Res.* 98 167–176. 10.1016/j.envres.2004.07.018 15820722

[B7] Bronfenbrenner U. (1979). *The ecology of human development: Experiments by nature and design.* Cambridge, MA: Harvard University Press.

[B8] Bronfenbrenner U. Ceci S. J. (1994). Nature-nurture reconceptualised: A bio-ecological model. *Psychol. Rev.* 10 568–586. 10.1037/0033-295X.101.4.568 7984707

[B9] Bronfenbrenner U. Evans G. W. (2000). Developmental science in 21st century: Emerging questions, theoretical models, research design and empirical findings. *Soc. Dev.* 9 115–125. 10.1111/1467-9507.00114

[B10] Bronfenbrenner U. Morris P. A. (1998). *The ecology of development processes.* New York, NY: John Wiley and Sons Inc.

[B11] Burris P. W. Phillips B. M. Lonigan C. J. (2019). Examining the relations of the home literacy environments of families of low ses with children’s early literacy skills. *J. Educ. Stud. Placed Risk* 24 154–173. 10.1080/10824669.2019.1602473 32346284 PMC7188069

[B12] Cameron C. E. Connor C. M. Morrison F. J. Jewkes A. M. (2008). Effects of classroom organization on letter-word reading in first grade. *J. Sch. Psychol.* 46 173–192. 10.1016/j.jsp.2007.03.002 19083356

[B13] Chiu Y. H. Hsu H. H. Coull B. A. Bellinger D. C. Kloog I. Schwartz J. (2016). Prenatal particulate air pollution and neurodevelopment in urban children: Examining sensitive windows and sex-specific associations. *Environ. Int.* 87 56–65. 10.1016/j.envint.2015.11.010 26641520 PMC4691396

[B14] Claudio L. Tulton L. Doucette J. Landrigan P. J. (1999). Socioeconomic factors and asthma hospitalization rates in New York City. *J. Asthma* 36 343–350. 10.3109/02770909909068227 10386498

[B15] Crosnoe R. Leventhal T. Wirth R. J. Pierce K. M. Pianta R. C. Nichd Early Child Care Research Network (2010). Family socioeconomic status and consistent environmental stimulation in early childhood. *Child Dev.* 81 972–987. 10.1111/j.1467-8624.2010.01446.x 20573117 PMC2892811

[B16] Davis A. N. Carlo G. Gulseven Z. Palermo F. Lin C. H. Nagel S. C. (2019). Exposure to environmental toxicants and young children’s cognitive and social development. *Rev. Environ. Health* 34 35–56. 10.1515/reveh-2018-0045 30844763

[B17] Demir O. E. Applebaum L. Levine S. C. Petty K. Goldin-Meadow S. (2011). “The story behind parent-child book-reading interactions: Specific relations to later language and reading outcomes,” in *Proceedings of the annual Boston university conference on language development*, Boston, MA. PMC3420826 22902865

[B18] Devlin J. T. Jamison H. L. Gonnerman L. M. Matthews P. M. (2006). The role of the posterior fusiform gyrus in reading. *J. Cogn. Neurosci.* 18 911–922. 10.1162/jocn.2006.18.6.911 16839299 PMC1524880

[B19] Dolean D. Melby-Lervåg M. Tincas I. Damsa C. Lervåge A. (2019). Achievement gap: Socioeconomic status affects reading development beyond language and cognition in children facing poverty. *Learn. Instr.* 63 1–10. 10.1016/j.learninstruc.2019.101218

[B20] Evans G. W. (2004). The environment of childhood poverty. *Am. Psychol.* 59 77–92. 10.1037/0003-066X.59.2.77 14992634

[B21] Federico M. J. Liu A. H. (2003). Overcoming childhood asthma disparities of the inner-city poor. *Pediatr. Clin. North Am.* 50 655–675. 10.1016/S0031-3955(03)00045-2 12877240

[B22] Flores G. Bauchner H. Feinstein A. R. Nguyen U. (1999). The impact of ethnicity, family income, and parental education on children’s health and use of health services. *Am. J. Public Health* 89 1066–1071. 10.2105/AJPH.89.7.1066 10394317 PMC1508855

[B23] Georgiou G. K. Tziraki N. Manolitsis G. Fella A. (2013). Is rapid automatized naming related to reading and mathematics for the same reason(s)? A follow-up study from kindergarten to grade 1. *J. Exp. Child Psychol.* 115 481–496. 10.1016/j.jecp.2013.01.004 23506806

[B24] Gershoff E. T. Aber J. L. Raver C. C. Lennon M. C. (2007). Income is not enough: Incorporating material hardship into models of income associations with parenting and child development. *Child Dev.* 78 70–95. 10.1111/j.1467-8624.2007.00986.x 17328694 PMC2835994

[B25] Grandjean P. Landrigan P. J. (2014). Neurobehavioural effects of developmental toxicity. *Lancet Neurol.* 13 330–338. 10.1016/S1474-4422(13)70278-3 24556010 PMC4418502

[B26] Grineski S. E. Collins T. W. Adkins D. E. (2020). Hazardous air pollutants are associated with worse performance in reading, math, and science among US primary school children. *Environ. Res.* 181:108925. 10.1016/j.envres.2019.108925 31776015

[B27] Guxens M. Lubczyńska M. J. Muetzel R. L. Dalmau-Bueno A. Jaddoe V. W. V. Hoek G. (2018). Air pollution exposure during fetal life, brain morphology, and cognitive function in school-age children. *Biol. Psychiatry* 84 295–303. 10.1016/j.biopsych.2018.01.016 29530279

[B28] Hajat A. Hsia C. O’Neill M. S. (2015). Socioeconomic disparities and air pollution exposure: A global review. *Curr. Environ. Health Rep.* 2 440–450. 26381684 10.1007/s40572-015-0069-5 PMC4626327

[B29] Han J. Neuharth-Pritchett S. (2015). Meaning-Related and print-related interactions between preschoolers and parents during shared book reading and their associations with emergent literacy skills. *J. Res. Child. Educ.* 29 528–550. 10.1080/02568543.2015.1073819

[B30] Hayes A. F. (2022). *The PROCESS macro for SPSS, SAS, and R.* Available online at: https://www.processmacro.org/download.html (accessed April 15, 2022).

[B31] Hensler B. S. Schatschneider C. Taylor J. Wagner R. K. (2010). Behavioral genetic approach to the study of dyslexia. *J. Dev. Behav. Pediatr.* 31 525–532. 10.1097/DBP.0b013e3181ee4b70 20814252 PMC2952936

[B32] Jbaily A. Zhou X. Liu J. Lee T. H. Kamareddine L. Verguet S. (2022). Air pollution exposure disparities across US population and income groups. *Nature* 601 228–233. 35022594 10.1038/s41586-021-04190-y PMC10516300

[B33] Kishiyama M. M. Boyce W. T. Jimenez A. M. Perry L. M. Knight R. T. (2009). Socioeconomic disparities affect prefrontal function in children. *J. Cogn. Neurosci.* 21 1106–1115. 10.1162/jocn.2009.21101 18752394

[B34] Lawson G. M. Hook C. J. Farah M. J. (2018). A meta-analysis of the relationship between socioeconomic status and executive function performance among children. *Dev. Sci.* 21 1–40. 10.1111/desc.12529 28557154 PMC5821589

[B35] Lertxundi A. Andiarena A. Martínez M. D. Ayerdi M. Murcia M. Estarlich M. (2019). Prenatal exposure to PM2.5 and NO2 and sex-dependent infant cognitive and motor development. *Environ. Res.* 174 114–121. 10.1016/j.envres.2019.04.001 31055169

[B36] Lewtas J. (1994). Human exposure to complex mixtures of air pollutants. *Toxicol. Lett.* 72 163–190. 10.1016/0378-4274(94)90024-8 8202929

[B37] Lovasi G. S. Treat C. A. Fry D. Shah I. Clougherty J. E. Berberian A. (2022). Clean fleets, different streets: Evaluating the effect of New York City’s clean bus program on changes to estimated ambient air pollution. *J. Expo. Sci. Environ. Epidemiol.* Advance online publication. 10.1038/s41370-022-00454-5 35906405 PMC10234802

[B38] Lu W. Hackman D. A. Schwartz J. (2021). Ambient air pollution associated with lower academic achievement among US children. *Environ. Epidemiol.* 5 1–25. 10.1097/EE9.0000000000000174 34909554 PMC8663889

[B39] Luthar S. S. Cushing G. (1999). *Measurement issues in the empirical study of resilience: An overview.* New York, NY: Kluwer Academic/Plenum Publishers.

[B40] Lyon G. R. Shaywitz S. E. Shaywitz B. A. (2003). A definition of dyslexia. *Ann. Dyslexia* 53 1–14.

[B41] Margolis A. E. Banker S. Pagliaccio D. De Water E. Curtin P. Bonilla A. (2020b). Functional connectivity of the reading network is associated with prenatal polybrominated diphenyl ether concentrations in a community sample of 5 year-old children: A preliminary study. *Environ. Int.* 134 1–22. 10.1016/j.envint.2019.105212 31743804 PMC7048018

[B42] Margolis A. E. Cohen J. W. Ramphal B. Thomas L. Rauh V. Herbstman J. (2022). Prenatal exposure to air pollution and early-life stress effects on hippocampal subregional volumes and associations with visuospatial reasoning. *Biol. Psychiatry Glob. Open Sci.* 2 292–300. 10.1016/j.bpsgos.2022.05.003 35978944 PMC9380862

[B43] Margolis A. E. Herbstman J. B. Davis K. S. Thomas V. K. Tang D. Wang Y. (2016). Longitudinal effects of prenatal exposure to air pollutants on self-regulatory capacities and social competence. *J. Child Psychol. Psychiatry* 57 851–860. 10.1111/jcpp.12548 26989990 PMC5333974

[B44] Margolis A. E. Ramphal B. Pagliaccio D. Banker S. Selmanovic E. Thomas L. V. (2021). Prenatal exposure to air pollution is associated with childhood inhibitory control and adolescent academic achievement. *Environ. Res.* 202:111570. 10.1016/j.envres.2021.111570 34181922 PMC8578437

[B45] Margolis A. E. Pagliaccio D. Davis K. S. Thomas L. Banker S. M. Cyr M. (2020a). Neural correlates of cognitive control deficits in children with reading disorder. Brain Imag. Behav. 14, 1531–1542. 10.1007/s11682-019-00083-x PMC6765445 30919230

[B46] Mayer S. E. Jencks C. (1989). Poverty and the distribution of material hardship. *J. Hum. Resour.* 24 88–114. 10.2307/145934

[B47] Messer D. Henry L. A. Nash G. (2016). The relation between executive functioning, reaction time, naming speed, and single word reading in children with typical development and language impairments. *Br. J. Educ. Psychol.* 86 412–428. 10.1111/bjep.12115 27106632

[B48] Mikati I. Benson A. F. Luben T. J. Sacks J. D. Richmond-Bryant J. (2018). Disparities in distribution of particulate matter emission sources by race and poverty status. *Am. J. Public Health* 108 480–485. 10.2105/AJPH.2017.304297 29470121 PMC5844406

[B49] Miller R. L. Garfinkel R. Horton M. Camann D. Perera F. P. Whyatt R. M. (2004). Polycyclic aromatic hydrocarbons, environmental tobacco smoke, and respiratory symptoms in an inner-city birth cohort. *Chest* 126 1071–1078. 10.1378/chest.126.4.1071 15486366 PMC2223076

[B50] Miranda M. L. Edwards S. E. Keating M. H. Paul C. J. (2011). Making the environmental justice grade: The relative burden of air pollution exposure in the United States. *Int. J. Environ. Res. Public Health* 8 1755–1771. 10.3390/ijerph8061755 21776200 PMC3137995

[B51] Montgomery L. E. Kiely J. L. Pappas G. (1996). The effects of poverty, race and family structure on US children’s health: Data from NHIS, 1978 through 1980 and 1989 through 1991. *Am. J. Public Health* 86 1401–1405. 10.2105/ajph.86.10.1401 8876508 PMC1380650

[B52] NCES (2020). *“Reading performance” the condition of education.* Available online at: https://nces.ed.gov/programs/coe/pdf/coe_cnb.pdf (accessed April 15, 2022).

[B53] New York City Department of Health (1998/1999). *Vital statistics.* New York, NY: New York City Department of Health.

[B54] Noble K. G. Farah M. J. McCandliss B. D. (2006a). Socioeconomic background modulates cognition-achievement relationships in reading. *Cogn. Dev.* 21 349–368. 10.1016/j.cogdev.2006.01.007 19789717 PMC2752890

[B55] Noble K. G. Wolmetz M. E. Ochs L. G. Farah M. J. McCandliss B. D. (2006b). Brain-behavior relationships in reading acquisition are modulated by socioeconomic factors. *Dev. Sci.* 9 642–654. 10.1111/j.1467-7687.2006.00542.x 17059461

[B56] Norton E. S. Wolf M. (2012). Rapid automatized naming (RAN) and reading fluency: Implications for understanding and treatment of reading disabilities. *Annu. Rev. Psychol.* 63 427–452. 10.1146/annurev-psych-120710-100431 21838545

[B57] Olden K. Poje J. (1995). Environmental justice and environmental health. *Bull. Soc. Occup. Environ. Health* 4 3–4.

[B58] Ouellette G. Beers A. (2008). A not-so-simple view of reading: How oral vocabulary and visual-word recognition complicate the story. *Read. Writ.* 23 189–208. 10.1007/s11145-008-9159-1

[B59] Ouellette T. Burstein N. Long D. Beecroft E. (2004). *Measures of material hardship: Final report.* Washington, DC: US Dept of Health and Human Services.

[B60] Padmanabhan V. Cardoso R. C. Puttabyatappa M. (2016). Developmental Programming, a pathway to disease. *Endocrinology* 157 1328–1340. 26859334 10.1210/en.2016-1003 PMC4816734

[B61] Pagliaccio D. Herbstman J. B. Perera F. Tang D. Goldsmith J. Peterson B. S. (2020). Prenatal exposure to polycyclic aromatic hydrocarbons modifies the effects of early life stress on attention and thought problems in late childhood. *J. Child Psychol. Psychiatry* 61 1253–1265. 10.1111/jcpp.13189 31907931 PMC7338249

[B62] Perera F. P. Illman S. M. Kinney P. L. Whyatt R. M. Kelvin E. A. Shepard P. (2002). The challenge of preventing environmentally related disease in young children: Community based research in New York City. *Environ. Health Perspect.* 110 197–204. 11836150 10.1289/ehp.02110197 PMC1240736

[B63] Perera F. P. Rauh V. Tsai W. Y. Kinney P. Camann D. Barr D. (2003). Effects of transplacental exposure to environmental pollutants on birth outcomes in a multiethnic population. *Environ. Health Perspect.* 111 201–205. 10.1289/ehp.5742 12573906 PMC1241351

[B64] Perera F. P. Rauh V. Whyatt R. M. Tsai W. Y. Tang D. Diaz D. (2006). Effect of prenatal exposure to airborne polycyclic aromatic hydrocarbons on neurodevelopment in the first 3 years of life among inner-city children. *Environ. Health Perspect.* 114 1287–1292. 16882541 10.1289/ehp.9084 PMC1551985

[B65] Perera F. P. Tang D. Wang S. Vishnevetsky J. Zhang B. Diaz D. (2012). Prenatal polycyclic aromatic hydrocarbon (PAH) exposure and child behavior at age 6-7 years. *Environ. Health Perspect.* 120 921–926. 10.1289/ehp.1104315 22440811 PMC3385432

[B66] Peterson B. S. Rauh V. A. Bansal R. Hao X. Toth Z. Nati G. (2015). Effects of prenatal exposure to air pollutants (polycyclic aromatic hydrocarbons) on the development of brain white matter, cognition, and behavior in later childhood. *JAMA Psychiatry* 72 531–540. 10.1001/jamapsychiatry.2015.57 25807066 PMC4456286

[B67] Peterson R. L. Pennington B. F. (2015). Developmental dyslexia. *Annu. Rev. Clin. Psychol.* 11 283–307. 10.1146/annurev-clinpsy-032814-112842 25594880

[B68] Price C. J. (2000). The anatomy of language: Contributions from functional neuroimaging. *J. Anat.* 197 335–359. 10.1046/j.1469-7580.2000.19730335.x 11117622 PMC1468137

[B69] Puglisi M. L. Hulme C. Hamilton L. G. Snowling M. J. (2017). The home literacy environment is a correlate, but perhaps not a cause, of variations in children’s language and literacy development. *Sci. Stud. Read.* 21 498–514. 10.1080/10888438.2017.1346660 29930486 PMC5972965

[B70] Rauh V. A. Whyatt R. M. Garfinkel R. Andrews H. Hoepner L. Reyes A. (2004). Developmental effects of exposure to environmental tobacco smoke and material hardship among inner-city children. *Neurotoxicol. Teratol.* 26 373–385. 10.1016/j.ntt.2004.01.002 15113599 PMC3376003

[B71] Raver C. C. Blair C. Willoughby M. (2013). Poverty as a predictor of 4-year-olds’ executive function: New perspectives on models of differential susceptibility. *Dev. Psychol.* 49 292–304. 10.1037/a0028343 22563675 PMC5460626

[B72] Richlan F. (2012). Developmental dyslexia: Dysfunction of a left hemisphere reading network. *Front. Hum. Neurosci.* 6:120. 10.3389/fnhum.2012.00120 22557962 PMC3340948

[B73] Richlan F. Kronbichler M. Wimmer H. (2009). Functional abnormalities in the dyslexic brain: A quantitative meta-analysis of neuroimaging studies. *Hum.Brain Mapp.* 30 3299–3308. 10.1002/hbm.20752 19288465 PMC2989182

[B74] Seidenberg M. S. Waters G. S. Barnes M. A. Tanenhaus M. K. (1984). When does irregular spelling or pronunciation influence word recognition? *J. Verbal Learn.Verbal Behav.* 23 383–404.

[B75] Shaywitz S. E. Shaywitz B. A. (2005). Dyslexia (specific reading disability). *Biol. Psychiatry* 57 1301–1309. 10.1016/j.biopsych.2005.01.043 15950002

[B76] Simos P. G. Breier J. I. Fletcher J. M. Foorman B. R. Castillo E. M. Papanicolaou A. C. (2002). Brain mechanisms for reading words and pseudowords: An integrated approach. *Cereb. Cortex* 12 297–305. 10.1093/cercor/12.3.297 11839603

[B77] Snowling M. J. Melby-Lervag M. (2016). Oral language deficits in familial dyslexia: A meta-analysis and review. *Psychol. Bull.* 142 498–545. 10.1037/bul0000037 26727308 PMC4824243

[B78] Stingone J. A. McVeigh K. H. Claudio L. (2017). Early-life exposure to air pollution and greater use of academic support services in childhood: A population-based cohort study of urban children. *Environ. Health* 16:2. 10.1186/s12940-017-0210-z 28100255 PMC5241986

[B79] Torgesen J. Wagner R. Rashotte C. (2012). *Test of word reading efficiency.* New York, NY: Pearson.

[B80] Vishnevetsky J. Tang D. Chang H. W. Roen E. L. Wang Y. Rauh V. (2015). Combined effects of prenatal polycyclic aromatic hydrocarbons and material hardship on child IQ. *Neurotoxicol. Teratol.* 49 74–80. 10.1016/j.ntt.2015.04.002 25912623 PMC4458400

[B81] Weinick R. M. Weigers M. E. Cohen J. W. (1998). Children’s health insurance, access to care, and health status: New findings. *Health Aff.* 17 127–136. 10.1377/hlthaff.17.2.127 9558790

[B82] Weiss B. (2000). Vulnerability of children and the developing brain to neurotoxic hazards. *Environ. Health Perspect.* 108 375–381. 10.1289/ehp.00108s3375 10852831 PMC1637834

[B83] Wodtke G. T. Parbst M. (2017). Neighborhoods, schools, and academic achievement: A formal mediation analysis of contextual effects on reading and mathematics abilities. *Demography* 54 1653–1676. 10.1007/s13524-017-0603-1 28755275

[B84] Wolf M. Bowers P. G. (1999). The double-deficit hypothesis for the developmental dyslexias. *J. Educ. Psychol.* 91 415–438. 10.1037/0022-0663.91.3.415

[B85] Woodcock R. McGrew M. Mather N. (2000). *Woodcock Johnson-III tests of achievement.* Rolling Meadows, IL: Riverside.

[B86] Yoshikawa H. Aber J. L. Beardslee W. R. (2012). The effects of poverty on the mental, emotional, and behavioral health of children and youth: Implications for prevention. *Am. Psychol.* 67 272–284. 10.1037/a0028015 22583341

[B87] Zaslow M. Bronte-Tinkew J. Capps R. Horowitz A. Moore K. A. Weinstein D. (2009). Food security during infancy: Implications for attachment and mental proficiency in toddlerhood. *Matern. Child Health* 13 66–80. 10.1007/s10995-008-0329-1 18317892

[B88] Zeki Al Hazzouri A. Elfassy T. Sidney S. Jacobs D. Pérez Stable E. J. Yaffe K. (2017). Sustained economic hardship and cognitive function: The coronary artery risk development in young adults study. *Am. J. Prev. Med.* 51 1–9. 10.1016/j.amepre.2016.08.009 27692543 PMC5167656

[B89] Zheng X. Li J. Li M. Wang Z. Cao X. Chen Y. (2022). Reduced vmPFC volume mediates the association between earlyexposure to family material hardship and problematic mobile phoneuse: The moderating role of parental attachment. *Curr. Psychol.* Advance online publication. 1–10. 10.1007/s12144-022-02720-z

