# Supplementary material for: Effects of prenatal polycyclic aromatic hydrocarbons and childhood material hardship on reading achievement in school-age children: A preliminary study
Source: Front Psychol. 2023 Jan 4;13:933177. doi: 10.3389/fpsyg.2022.933177 (PMC9845780; doi:10.3389/fpsyg.2022.933177)
Supplement: Supplementary file 1 [file Data_Sheet_1.docx]

**Supplementary Methods**

**Polycyclic Aromatic Hydrocarbon (PAH) Assessment**

As described elsewhere (Perera 2003), polyurethane foam cartridges housed in air monitoring backpacks were analyzed at Southwest Research Institute in San Antonio, Texas, for concentrations of eight carcinogenic PAHs. Particle bound and volatile and semi-volatile PAH were extracted from the filter and PUF via a Soxhlet Extractor and extracts were assayed by GC-MS for pyrene and eight non-volatile, carcinogenic PAH: benz[*a*]anthracene, chrysene, benzo[*b*]fluroanthene, benzo[*k*]fluroanthene, B[a]P, indeno[1,2,3-*cd*]pyrene, dibenz[*a,h*]anthracene and benzo[*g,h,i*]perylene. For analyses, exposure to airborne PAH is expressed as the natural log of the sum of the concentrations of the eight PAH. Values below the level of detection (LOD) were calculated as LOD/2. Scores are reported as ng/m^3^. Women all wore the backpack in third trimester of pregnancy continuously for 48 hours. The PAH value is standardized for the number of hours worn per person.

**Supplementary Figures**

**Figure S1.** Test of Normality Q-Q Plots of WJ-III Basic Reading Index and TOWRE Word Reading Efficiency Index

Normality Q-Q plots for reading indexes assessed in main analyses. (A) Shows Q-Q plot for TOWRE-Word Reading Efficiency Index and (B) shows Q-Q plot for WJ-III Basic Reading Index.


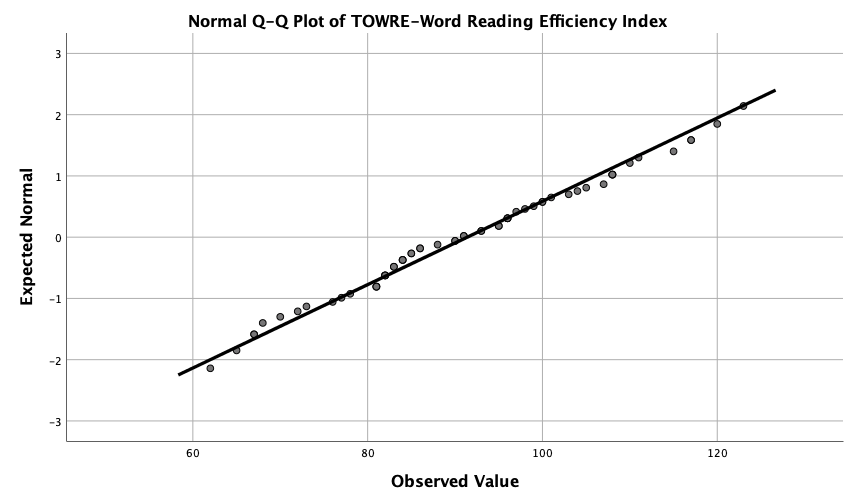

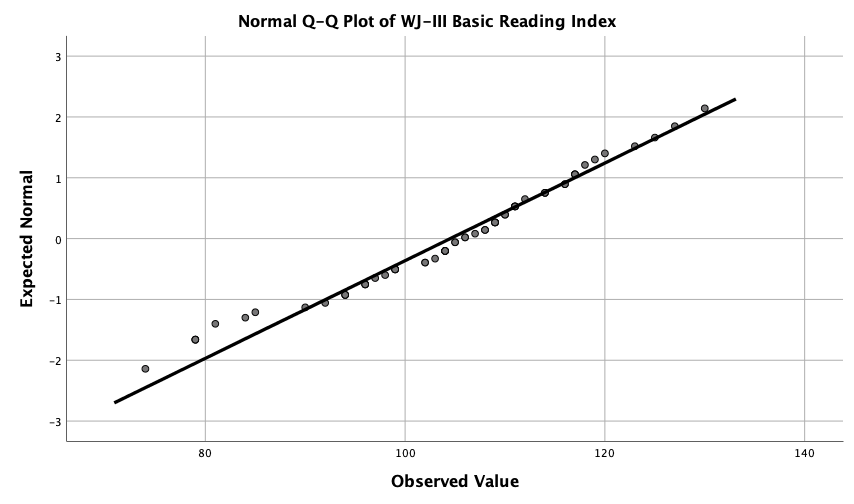


**A.**

**B.**

**Figure S2.** Test of Normality Q-Q Plots of prenatal PAH and Material Hardship


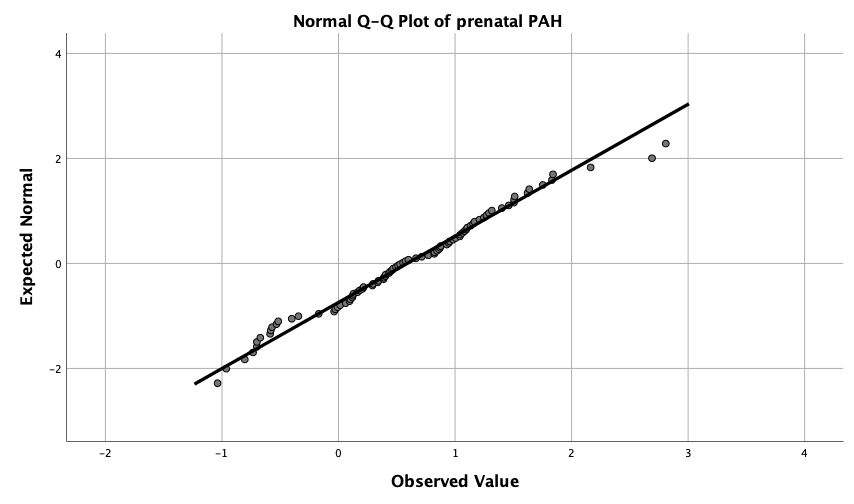


**A.**

**B.**


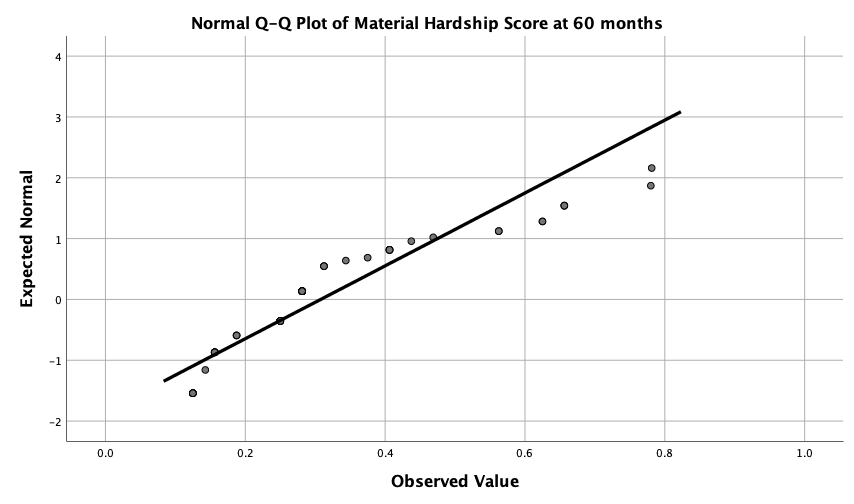


Normality Q-Q plots of predictors assessed in main analyses. (A) Shows Q-Q plot for prenatal PAH and (B) shows Q-Q plot for Material Hardship.

**Figure S3.** Probability-Probability plots of regression standardized residual reading measures

**A.**


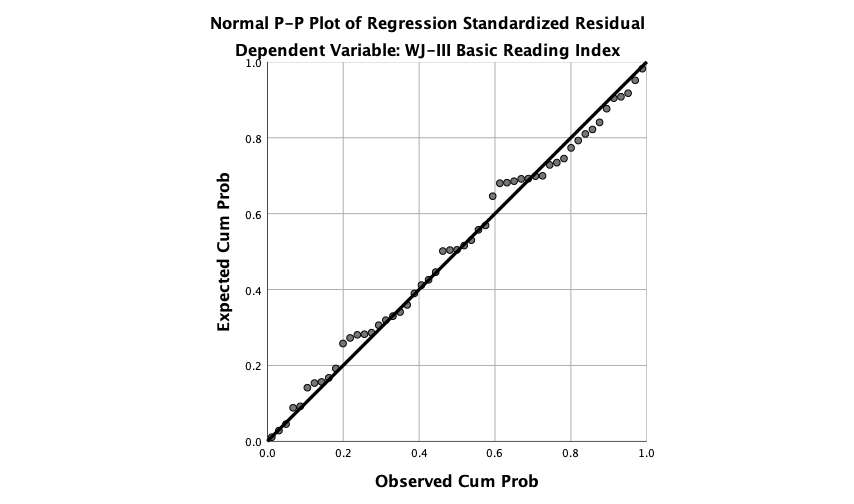


**B.**


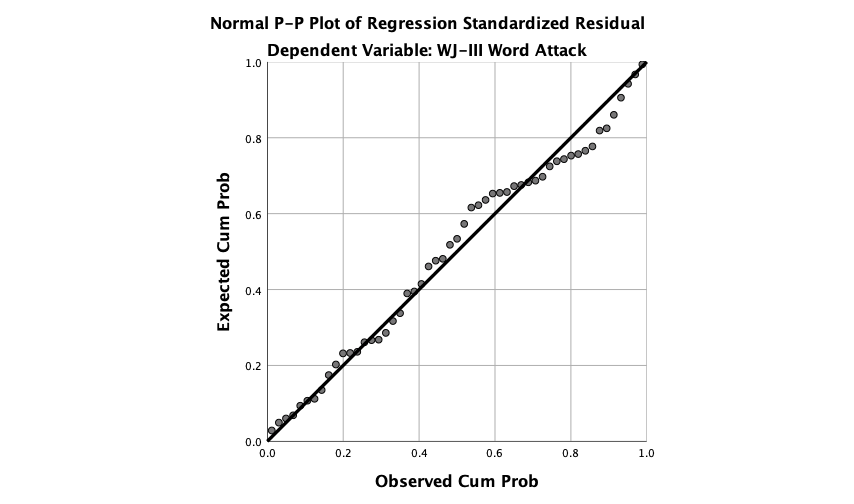


**Figure S3.** Probability-probability (P-P) plots of significant results from multiple linear regression analyses. P-P plots of sex, birthweight (grams), ethnicity/race, maternal education (prenatal), smoker in the house (prenatal), material hardship and prenatal PAH exposure regressed onto A) WJ-III Basic Reading Index, B) Word Attack standard score.

**Supplementary Tables**

**Table S1.** Geometric mean, median and range for 8 polycyclic aromatic hydrocarbons

| **N=53** | **8 Polycyclic Aromatic Hydrocarbons (ng/m^3^)** |  |
| --- | --- | --- |
|  | **Geometric Mean [median]** | **Range** |
| **benz[a]anthracene (baa2)** | 0.168[0.160] | 0.03-0.60 |
| **benzo[a] pyrene (B[a]P)** | 0.251[0.273] | 0.04-1.69 |
| **chrysene** | 0.171[0.178] | 0.03-0.92 |
| **benzo [b]fluoranthene** | 0.242[0.282] | 0.04-0.94 |
| **benzo[k] fluoranthene** | 0.077[0.050] | 0.03-0.28 |
| **indeno [1,2,3-cd]pyrene** | 0.257[0.274] | 0.04-1.40 |
| **dibenz[a,h]anthracene** | 0.044[0.043] | 0.01-0.11 |
| **benzo[g,h,i]perylene** | 0.501[0.570] | 0.08-4.83 |
| **Total PAH** | 1.796[1.823] | 0.35-8.70 |
| **Natural Log of PAH** | [0.601] | -1.04-2.16 |

**Table S2.** Effects of prenatal PAH exposure at values material hardship on WJ-III Basic Reading Index

| Z-Score Material Hardship | Effect | Standard Error | T-score | P-value | LLCI | ULCI |
| --- | --- | --- | --- | --- | --- | --- |
| -0.9369 | 3.4909 | 2.9599 | 1.1794 | 0.2446 | -2.4745 | 9.4563 |
| -0.1595 | -1.2054 | 2.1852 | -0.5516 | 0.5840 | -5.6095 | 3.1987 |
| 0.8436 | -7.2648 | 3.6244 | -2.0044 | 0.0512 | -14.5693 | 0.0397 |

The table displays conditional effects of prenatal PAH exposure at values of material hardship on WJ-III Basic reading Index. For every 1 point increase in prenatal PAH exposure at high levels of material hardship, there is a 7 point decrease in WJ-III Basic Reading Index. Significant interaction terms (p<.05).

**Table S3.** Primary regression analyses with WJ-III Letter Word Identification and Word Attack, as dependent variables

|  | WJ-III Letter Word Identification  (n=53) | | | WJ-III  Word Attack  (n=53) | | |
| --- | --- | --- | --- | --- | --- | --- |
|  | β | t | P | β | t | P |
| Ethnicity/race | -.014 | -.102 | .919 | .195 | 1.467 | .149 |
| Maternal education (prenatal) | .020 | .126 | .900 | -.128 | -.827 | .412 |
| Sex | -.063 | -.426 | .672 | -.100 | -.707 | .483 |
| Birth weight (grams) | -.304 | -1.964 | .056 | -.267 | -1.812 | .077 |
| Smoker in the house (prenatal) | .010 | .061 | .952 | -.057 | -.366 | .716 |
| Ln total PAH (prenatal) | -.088 | -.593 | .556 | -.200 | -1.420 | .163 |
| Material Hardship at age 5 | .097 | .596 | .554 | .022 | .145 | .885 |
| PAH x Material Hardship | -.279 | -1.731 | .090 | -.391 | -2.550 | .014 |

Full regression analyses are presented for WJ-III Letter Word Identification and Word Attack. Multiple linear regressions were analyzed to determine if prenatal PAH exposure and material hardship at age 5 predicted reading skill level at age 7, controlling for ethnicity/race, maternal education (prenatal), sex, birthweight (grams), and smoker in the house (prenatal). Standardized regression coefficients (**β**) and their corresponding t-statistic and p-values are presented for all predictors on the table. Significant interaction terms (p<.05).

**Table S4.** Model including age as a covariate on the effects of prenatal PAH exposure and material hardship on WJ-III Basic Reading Index

| **N=53** | **WJ-III Basic Reading Index** | | |
| --- | --- | --- | --- |
|  | **β** | **t** | **P** |
| **Age** | -0.170 | -1.198 | 0.237 |
| **Ethnicity/Race** | 0.089 | 0.654 | 0.517 |
| **Maternal education (prenatal)** | -0.045 | -0.287 | 0.776 |
| **Sex** | -0.123 | -0.831 | 0.410 |
| **Birth weight (grams)** | -0.264 | -1.718 | 0.093 |
| **Smoker in the house (prenatal)** | -0.050 | -0.305 | 0.761 |
| **Ln total PAH (prenatal) – Z-scored** | -0.160 | -1.099 | 0.278 |
| **Material Hardship at age 5 – Z-scored** | 0.036 | 0.224 | 0.824 |
| **PAH x Material Hardship *** | **-0.348** | **-2.219** | **0.032** |

**Table S5.** Effects of prenatal PAH exposure at values material hardship on WJ-III Word Attack

| Z-Score Material Hardship | Effect | Standard Error | T-score | P-value | LLCI | ULCI |
| --- | --- | --- | --- | --- | --- | --- |
| -.9369 | 2.8989 | 2.5491 | 1.1372 | .2616 | -2.2385 | 8.0364 |
| -.1595 | -1.7952 | 1.8819 | -.0539 | .3453 | -5.5880 | 1.9977 |
| .8436 | -7.8516 | 3.1213 | -2.5155 | -.0156 | -14.1424 | -1.5609 |

The table displays conditional effects of prenatal PAH exposure at values of material hardship on WJ-III Word Attack. For every 1 point increase in prenatal PAH exposure at high levels of material hardship, there is a 7 point decrease in WJ-III Word Attack. Significant interaction terms (p<.05).
